# Identification TRIM46 as a Potential Biomarker and Therapeutic Target for Clear Cell Renal Cell Carcinoma Through Comprehensive Bioinformatics Analyses

**DOI:** 10.3389/fmed.2021.785331

**Published:** 2021-11-22

**Authors:** Xiang-bin Ren, Jing Zhao, Xue-feng Liang, Xu-dong Guo, Shao-bo Jiang, Yu-zhu Xiang

**Affiliations:** ^1^Department of Urology, Shandong Provincial Hospital, Cheeloo College of Medicine, Shandong University, Jinan, China; ^2^Department of Orthopaedics, Shandong Provincial Hospital Affiliated to Shandong First Medical University, Jinan, China; ^3^Department of Blood Supply, Shandong Blood Center, Jinan, China

**Keywords:** TRIM46, clear cell renal cell carcinoma, prognosis, biomarker, therapeutic target

## Abstract

**Background:** Tripartite motif containing 46 was initially identified as the oncogene in several human tumors. However, the clinical value and potential functions of tripartite motif containing 46 (TRIM46) in clear cell renal cell carcinoma (ccRCC) remained largely unclear.

**Methods:** The expressing patterns, clinical involvement, and prognostic values of TRIM46 were analyzed using the data obtained from TCGA and GEO databases. A nomogram was constructed to examine the outcome of patients with ccRCC. We estimated the association between TRIM46 with tumor immunity in ccRCC.

**Results:** Tripartite motif containing 46 was highly expressed in ccRCC, and its upregulation revealed an unfavorable prognosis. A nomogram based on TRIM46 expressions and other independent prognostic factors could robustly predict the overall survival of tumor patients. TRIM46 has a strong positive correlation with NUMBL, CACNB1, THBS3, ROBO3, MAP3K12, ANKRD13D, PIF1, PRELID3A, ANKRD13B, and PCNX2. Mechanically, TRIM46 displayed regulatory functions in ccRCC progression *via* several tumor-associated pathways. Besides, we observed that TRIM46 was distinctly related to tumor immunity in ccRCC.

**Conclusions:** Our findings provide a novel tumor promotive role regarding TRIM46 function in the malignant progression of ccRCC.

## Introduction

Renal cell carcinoma is one of the most common urinary malignancies worldwide and its incidence is gradually increasing (1). Clear cell renal cell carcinoma (ccRCC) accounts for 70–85% of all renal tumors (2). Metastasis and recurrence are important biological characteristics, leading to a poor prognosis (3). With the development of immunotherapy and targeted therapy, a novel epoch may arrive. However, the existing treatments have several inferiorities, including drug resistance, higher systemic toxicity, and, in some cases, incidence rate and mortality (4, 5). In recent decades, the prognostic value of novel biomarkers has become increasingly attractive. The identification of novel sensitive biomarkers is eager for the optimization of the treatment plan.

Tripartite motif containing 46, a member of the family of tripartite motif (TRIM)-containing proteins, was defined as an essential modulator of oncogenesis in several malignancies (6–8). In osteosarcoma, deletion of tripartite motif containing 46 (TRIM46) suppresses the activity of tumor cells, inhibits cellular cycle, and induces cellular apoptosis, whereas upregulation of TRIM46 displays the opposite effect (9). In breast cancer, TRIM46 acts as a positive regulator in the proliferation and migration of tumor cells (10). Although TRIM46 has demonstrated its potential as a biomarker in cancers, its clinical value and specific effects on the occurrence and progression of ccRCC remained largely unclear.

In this study, we analyzed the TCGA and GEO databases to determine the expressing pattern of TRIM46 and its prognostic value in ccRCC. Then, we performed functional enrichment analyses, including Gene Ontology (GO), Kyoto Encyclopedia of Genes and genomes (KEGG0, and Gene Set Enrichment Analysis (GSEA), to delve into the possible mechanisms involved in TRIM46 function. Moreover, the association of TRIM46 and tumor immunity was equally uncovered in this study.

## Materials and Methods

### Data Collection

The transcriptomic profiles were downloaded from the TCGA datasets (11). After we excluded those samples with incomplete clinical information, this study included 530 ccRCC specimens and 72 non-tumor renal samples. Clinicopathological characteristics of patients with ccRCC were presented in Supplementary Table 1. Besides, the data from the GSE36895, GSE66272, and GSE53757 datasets were applied to further confirm the results of TCGA datasets.

### Functional Enrichment Analysis

Metascape was applied to perform functional enrichment assays of TRIM46 and its related genes (12). Moreover, GSEA was utilized to investigate the possible mechanisms of TRIM46 in ccRCC. Gene sets with *p* < 0.05 were considered a significant enrichment.

### Relationship of TRIM46 With Tumor-Infiltrating Immune Cells (TIICs) in CcRCC

In this study, the “Gene” module was applied to examine the association between TRIM46 expressions and six immune cell infiltration levels. CIBERSORT is a free experimental database that can measure the composition of the immune cells in tissues according to their gene expression profile (13). After the expression profile of the TCGA-KIRC, dataset with standard gene annotation was uploaded to the CIBERSORT portal, the LM22 signature, and permutation = 100 deconvolution algorithm served to calculate the immune cell infiltration. The CIBERSORT value generated was defined as the fraction of immune cell infiltration in each sample. Then, our group analyzed the differences in the content of various immune infiltrating cells between low- and high-expressing groups. Additionally, the association of TRIM46 and immunosuppressive molecules, including PDCD1, LAG3, CTLA4, CD276, and TIGIT, was analyzed in the TCGA database.

### Statistical Analysis

The R 3.6.1 software (The R Foundation for Statistical Computing, Vienna, Austria) was used for all statistical analyses. The prognostic values of TRIM46 in ccRCC were determined by the use of Kaplan-Meier survival method, followed by log-rank test. The independent prognostic factors were identified by the use of uni- and multivariate analyses. Spearman correlation analysis was used to analyze the relationship between TRIM46 and immunosuppressive molecules. The difference was considered significant when *p* < 0.05.

## Results

### The Distinct Upregulation of TRIM46 in CcRCC

**Figure 1:**
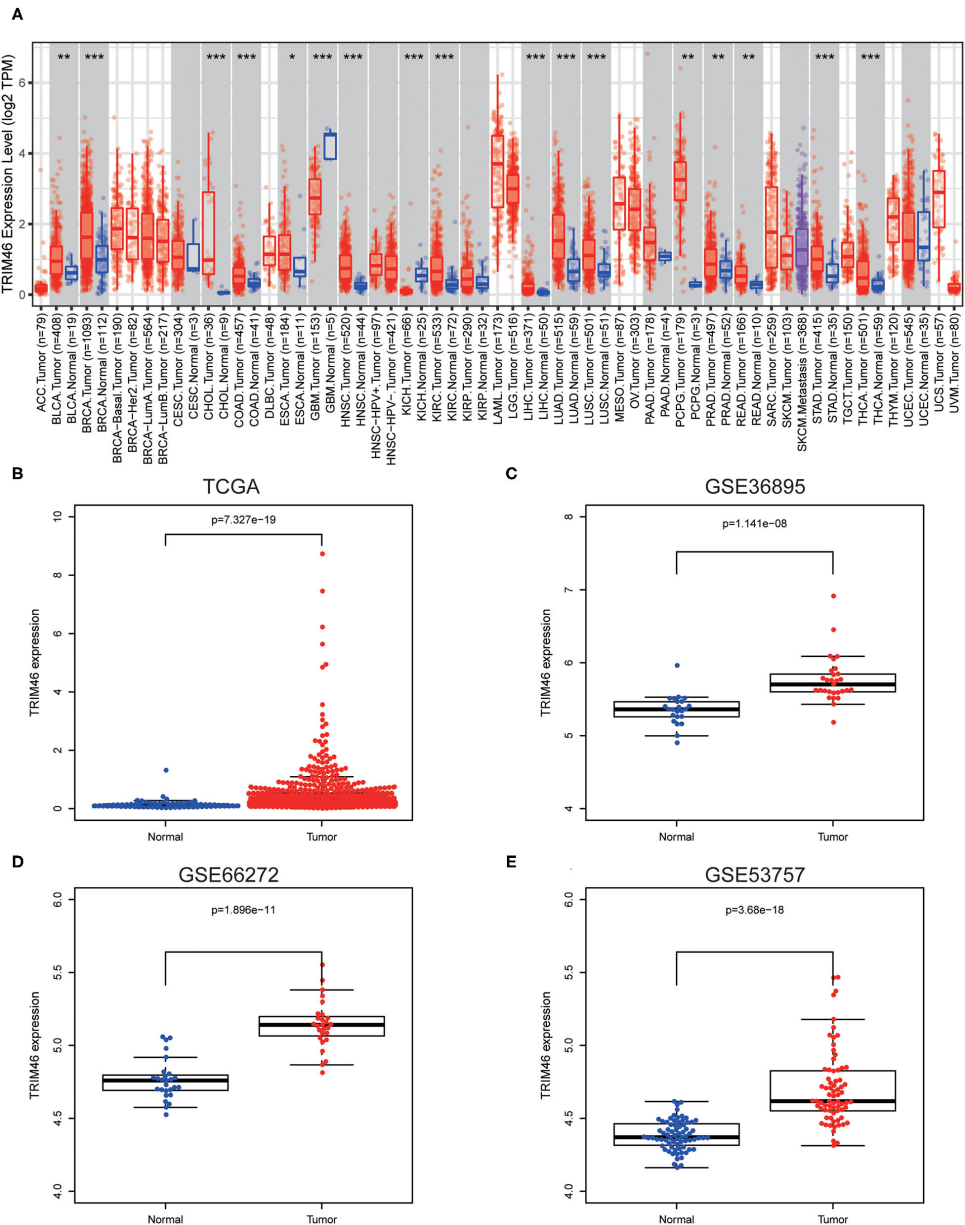
The distinct upregulation of tripartite motif containing 46 (TRIM46) in clear cell renal cell carcinoma (ccRCC) specimens. **(A)** TRIM46 expressions in several types of tumors *via* analyzing TCGA datasets were confirmed by the use of TIMER. **(B–E)** The overexpression of TRIM46 was demonstrated *via* analyzing data from TCGA, GSE36895, GSE66272, and GSE53757. ^*^p < 0.05, ^**^p < 0.01, ^***^p < 0.001.

Firstly, we analyzed the TIMER database, finding that TRIM46 exhibited an increased level in many types of tumors, including LIHC, KIRC, KICH, READ, PRAD, THCA, STAD, ESCA, COAD, CHOL, HNSC, BRCA, BLCA PCPG, LUSC, and LUAD (Figure 1A). However, a decreased expression of TRIM46 was observed in GBM. The data from TCGA, GSE36895, GSE66272, and GSE53757 also indicated that TRIM46 was highly expressed in ccRCC relative to normal tissue controls (both *p* < 0.001) (Figures 1B–E).

### The Association Between TRIM46 Expression With Clinicopathologic Variables

**Figure 2:**
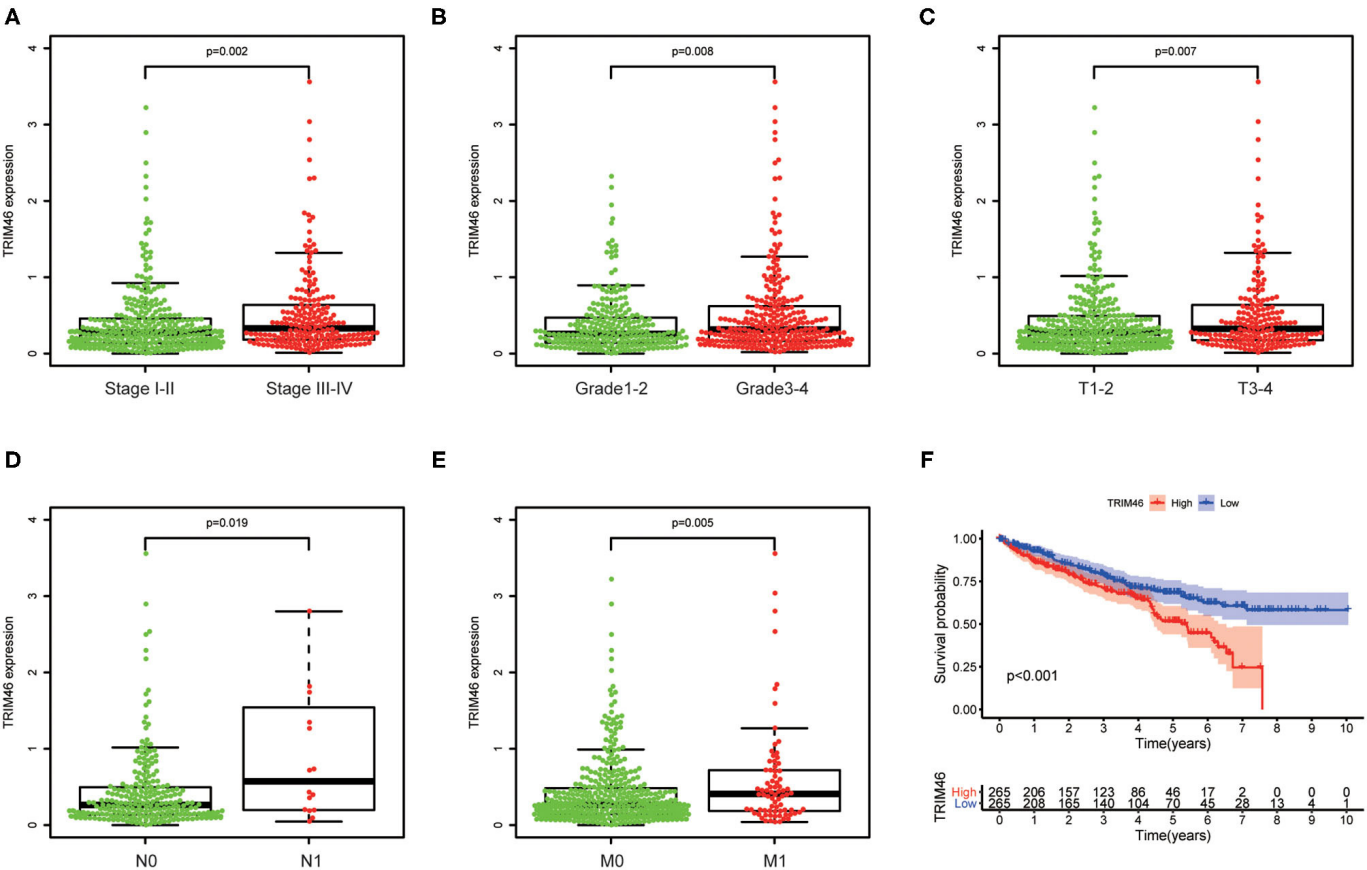
The relationship between TRIM46 and clinical features. **(A)** Stage; **(B)** grade; **(C)** T stage; **(D)** N stage; **(E)** M stage. **(F)** Kaplan-Meier survival analysis.

We next investigate the link between the expression levels of TRIM46 with clinical features of patients with ccRCC. We found that high TRIM46 expression was associated with high clinical stage (*p* = 0.002, Figure 2A), histologic grade (*p* = 0.008, Figure 2B), T classification (Figure 2C), N classification (Figure 2D), and M classification (Figure 2E).

### TRIM46 Overexpression Indicates a Worse Survival in CcRCC

**Figure 3:**
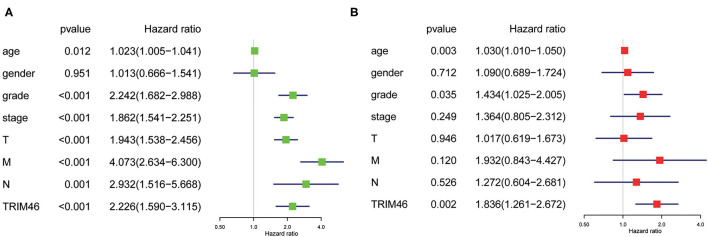
**(A)** Univariate Cox regression analysis. **(B)** Multivariate Cox regression analysis.

Then, we used the Kaplan-Meier survival curves to assess the prognostic values of TRIM46 in ccRCC. Kaplan-Meier assay results indicated that high TRIM46 expression subgroup had a distinctly shorter overall survival (*p* < 0.001) (Figure 2F). Moreover, the uni- and multivariate analyses suggested that TRIM46 expression (*p* = 0.002; HR = 1.836), age (*p* = 0.003; HR = 1.030), and histologic grade (*p* = 0.035; HR = 1.434) were independent biomarkers for overall survival (OS) of ccRCC (Figures 3A,B). Collectively, TRIM46 is a potential independent risk factor in ccRCC.

### A Nomogram Was Developed to Predict OS of CcRCC

**Figure 4:**
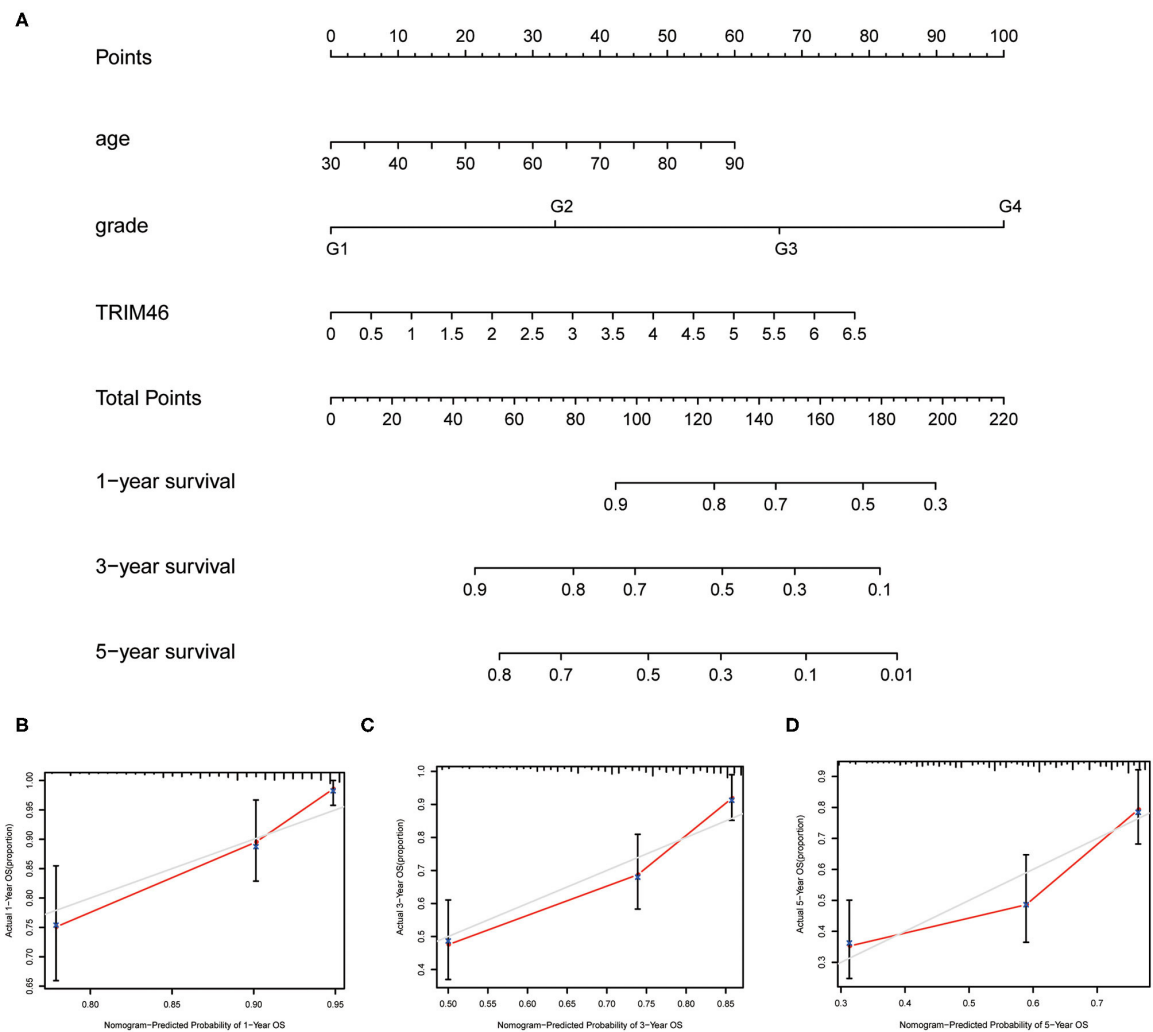
The prognostic value of several factors in a nomogram. **(A)** Construction of a predictive nomogram. **(B–D)** Actual and predicted survivals were shown by the use of calibration curves.

To better predict the long-term survivals of patients, we used the above independent of constructing nomogram to predict the long-term survivals of patients with ccRCC (Figure 4A). Importantly, the calibration curves revealed that the actual and predicted survival matched very well in terms of 1-year (Figure 4B), 3-year (Figure 4C), and 5-year OS (Figure 4D).

### Co-expression and Functional Enrichment Analysis

**Figure 5:**
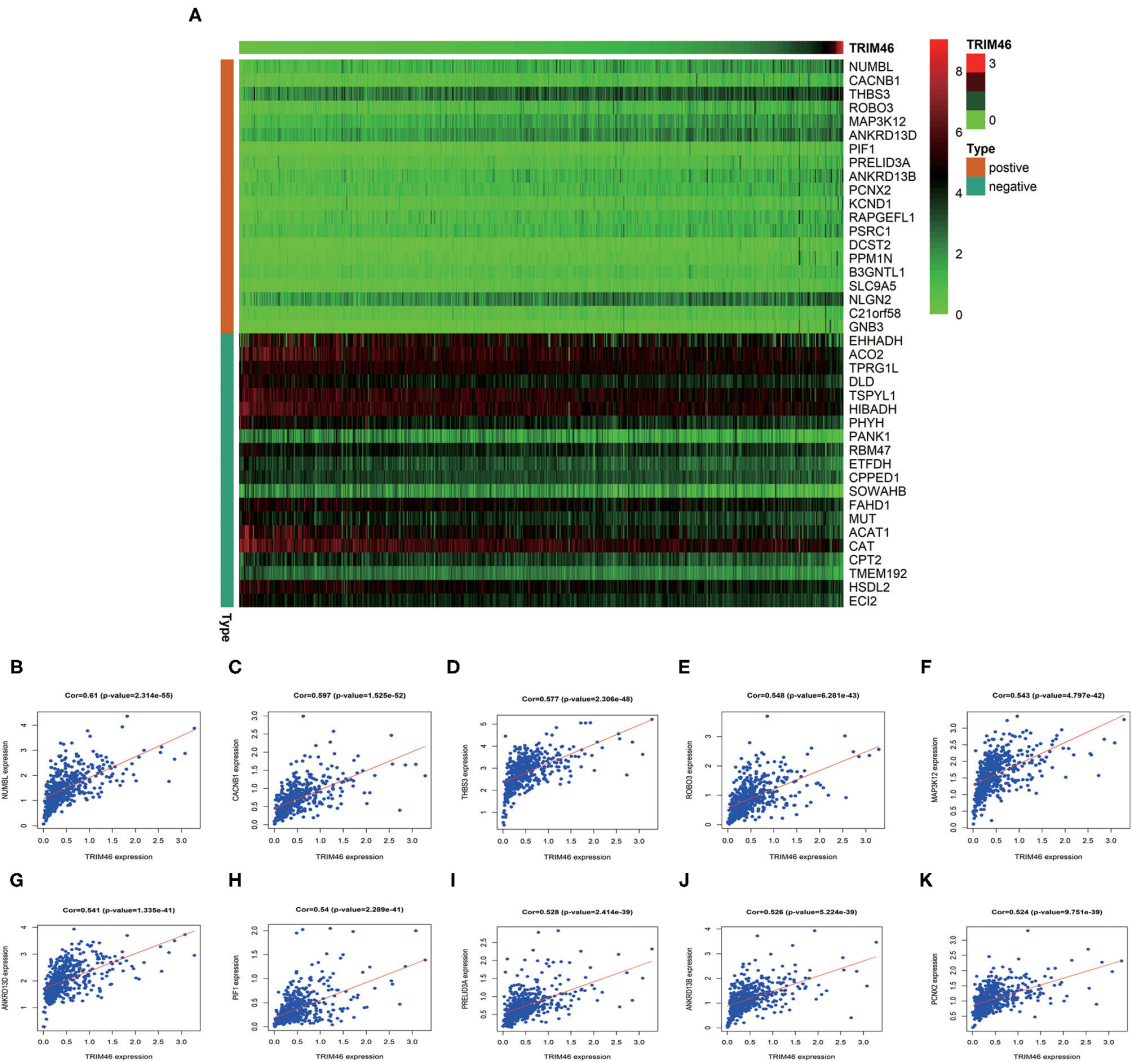
Co-expression assays. **(A)** The top 20 genes related to TRIM46. **(B–K)** The association of TRIM46 with NUMBL, CACNB1, THBS3, ROBO3, MAP3K12, ANKRD13D, PIF1, PRELID3A, ANKRD13B, and PCNX2.

**Figure 6:**
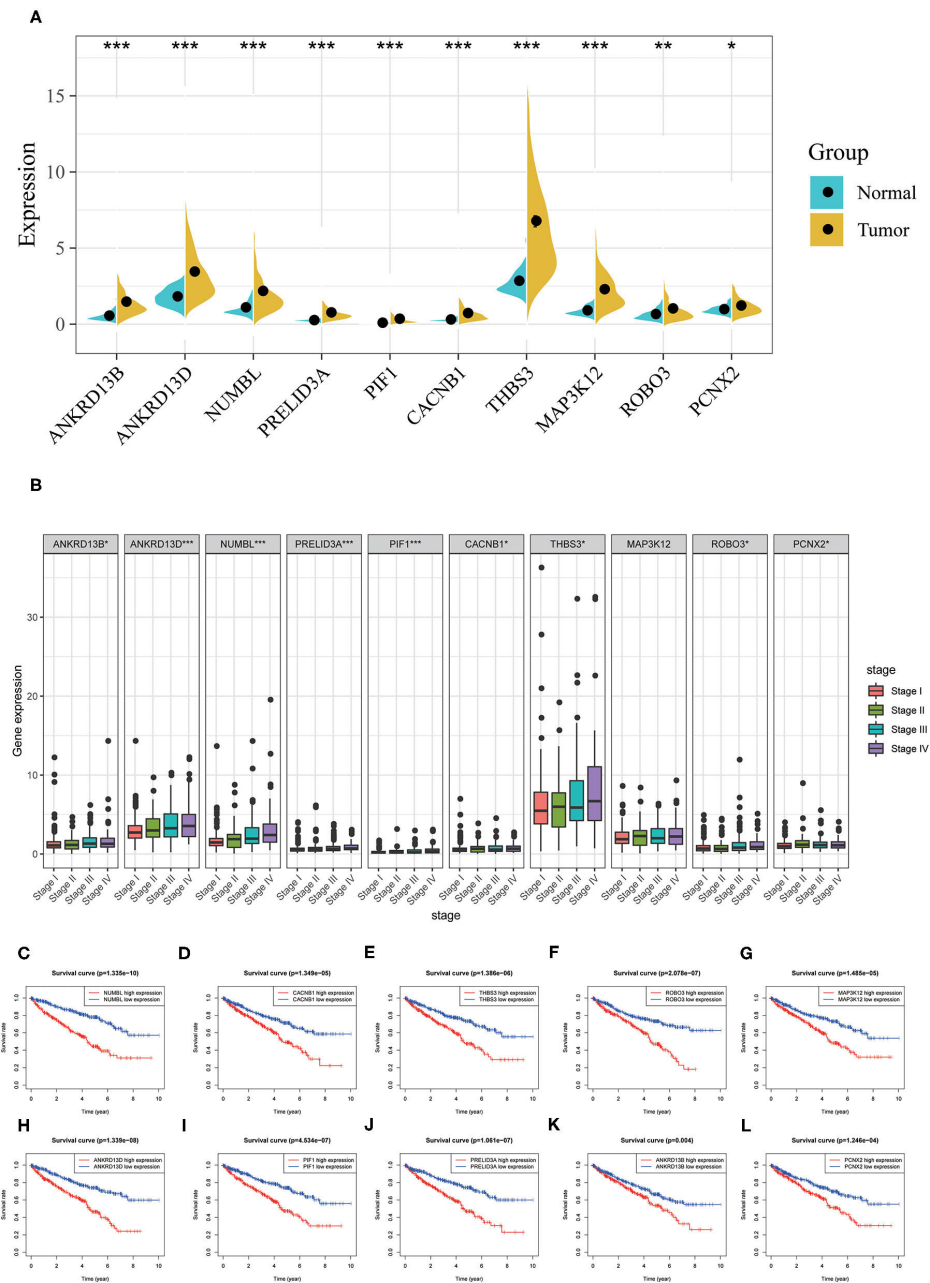
Analyses of co-expressed genes with TRIM46. **(A)** NUMBL, CACNB1, THBS3, ROBO3, MAP3K12, ANKRD13D, PIF1, PRELID3A, ANKRD13B, and PCNX2 were overexpressed in ccRCC; **(B)** higher levels of NUMBL, CACNB1, THBS3, ROBO3, MAP3K12, ANKRD13D, PIF1, PRELID3A, ANKRD13B, and PCNX2 indicated advanced clinical stage for patients with ccRCC; **(C–L)** higher expression level of NUMBL, CACNB1, THBS3, ROBO3, MAP3K12, ANKRD13D, PIF1, PRELID3A, ANKRD13B, and PCNX2 indicated worse OS for patients with ccRCC (^*^p < 0.05, ^**^p < 0.01, ^***^p < 0.001).

**Figure 7:**
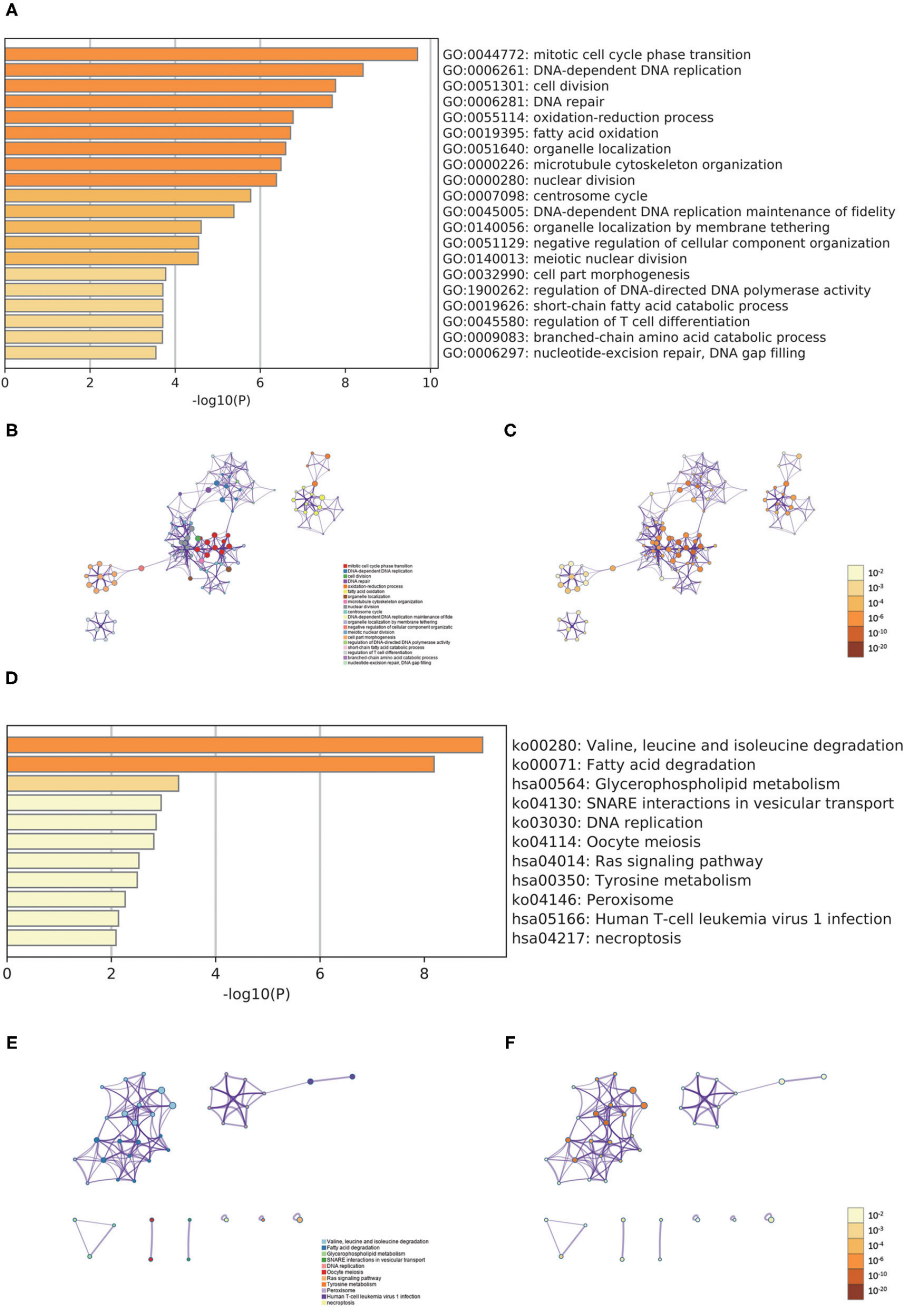
Functional enrichment analyses. **(A)** Heatmap of the biological processes enriched terms; **(B)** network of biological processes enriched terms; **(C)** network of biological processes enriched terms; **(D)** heatmap of the pathways enriched terms; **(E)** network of pathways enriched terms; **(F)** network of pathways enriched terms.

In order to better examine the possible meaning of TRIM46 in ccRCC, we first performed the co-expression analysis of TRIM46; 382 genes were chosen for further analysis (Supplementary Table 2). Moreover, Figure 5A shows the heatmaps of the top 20 genes related with TRIM46. Additionally, TRIM46 has a strong positive correlation with NUMBL, CACNB1, THBS3, ROBO3, MAP3K12, ANKRD13D, PIF1, PRELID3A, ANKRD13B, and PCNX2 (both *p* < 0.001) (Figures 5B–K). We, thus, explored their expression patterns, clinical significance, and prognostic value in ccRCC using the TCGA-KIRC data. Interestingly, higher expression levels of NUMBL, CACNB1, THBS3, ROBO3, MAP3K12, ANKRD13D, PIF1, PRELID3A, ANKRD13B, and PCNX2 were found in ccRCC samples (Figure 6A), and their overexpression was all associated with advanced stage (Figure 6B) and poorer OS (Figures 6C–L), indicating TRIM46 and its 10 functional partners might be involved in ccRCC progression together. GO_BP assays revealed that TRIM46 and the related genes were mainly enriched in biological processes linked to mitotic cell cycle phase transition, DNA-dependent DNA replication, and cell division (Figures 7A–C). KEGG assays revealed that TRIM46 and its partners were distinctly enriched in valine, leucine, and isoleucine degradation; fatty acid degradation; and glycerophospholipid metabolism (Figures 7D–F), suggesting that TRIM46 and its functional partners might influence ccRCC progression by regulating cell proliferation and substance metabolism.

### TRIM46-Related Signaling Pathways

**Figure 8:**
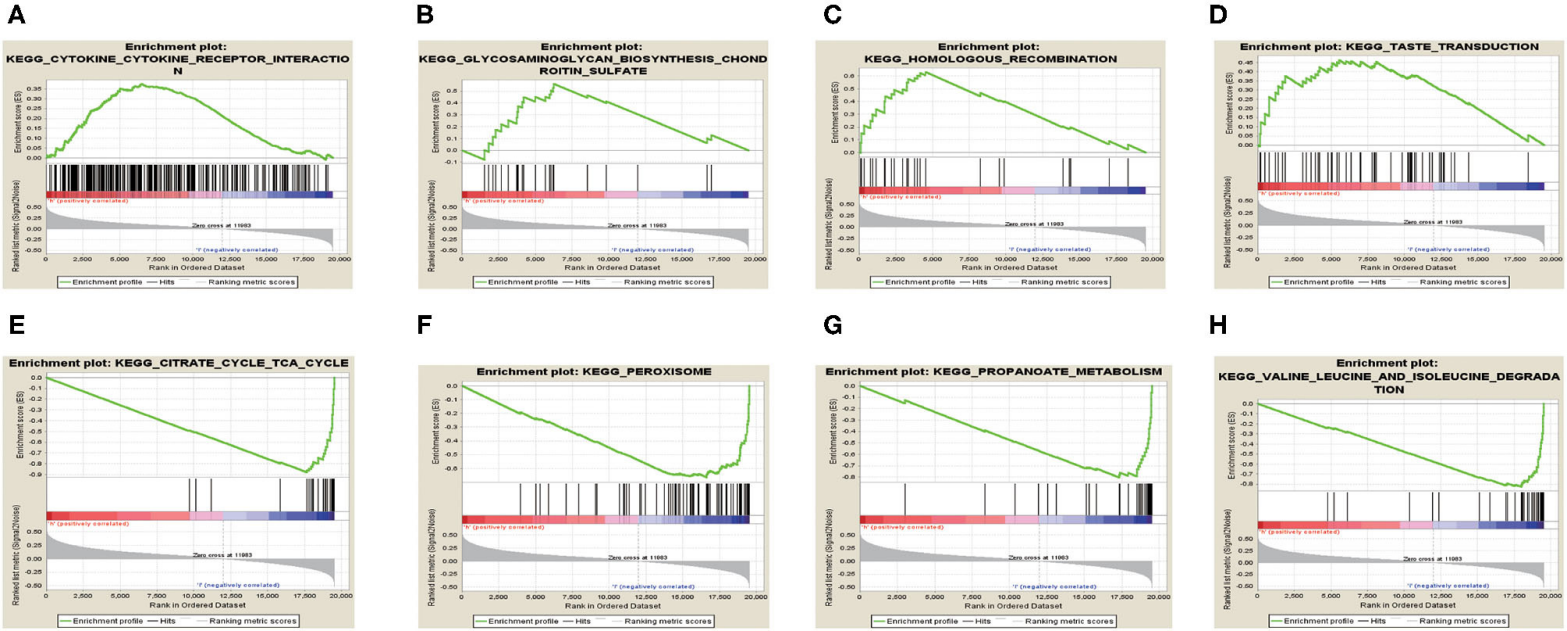
Gene set enrichment analysis. **(A–D)** The four most significantly enriched signaling pathways enriched in the high TRIM46 expression phenotype, respectively. **(E–H)** The four most significantly enriched signaling pathways enriched in the low TRIM46 expression phenotype, respectively.

We performed GSEA analysis and results showed that in the high TRIM46 expression phenotype, the four most significantly enriched signaling pathways were the cytokine cytokine–receptor interaction, chondroitin sulfate biosynthesis, taste transduction, and homologous recombination (Figures 8A–D). Whereas, in the low TRIM46 expression phenotype, the four most significantly enriched signaling pathways were citrate cycle TCA cycle, peroxisome, propanoate metabolism and valine, and leucine and isoleucine degradation (Figures 8E–H).

### Relationship Between TRIM46 and TIICs, Immunosuppressive Molecules

**Figure 9:**
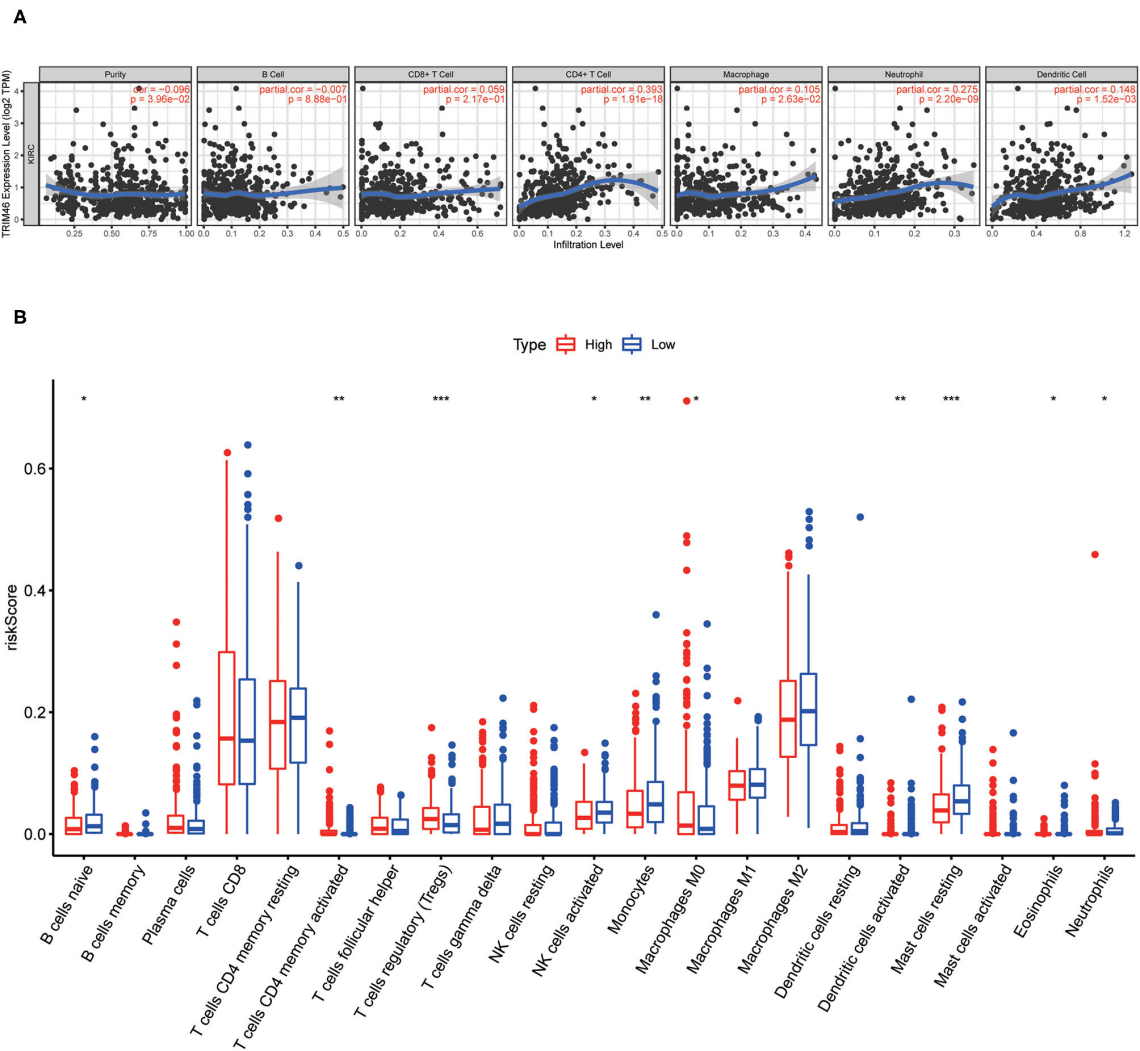
Associations of tumor-infiltrating immune cell (TIIC) proportion with TRIM46 expressions. **(A)** Six immune cell infiltration levels were involved in the expressions of TRIM46 in in ccRCC (TIMER database); **(B)** barplot of the proportion of 21 kinds of TIICs in ccRCC specimens (^*^p < 0.05, ^**^p < 0.01, ^***^p < 0.001).

**Figure 10:**
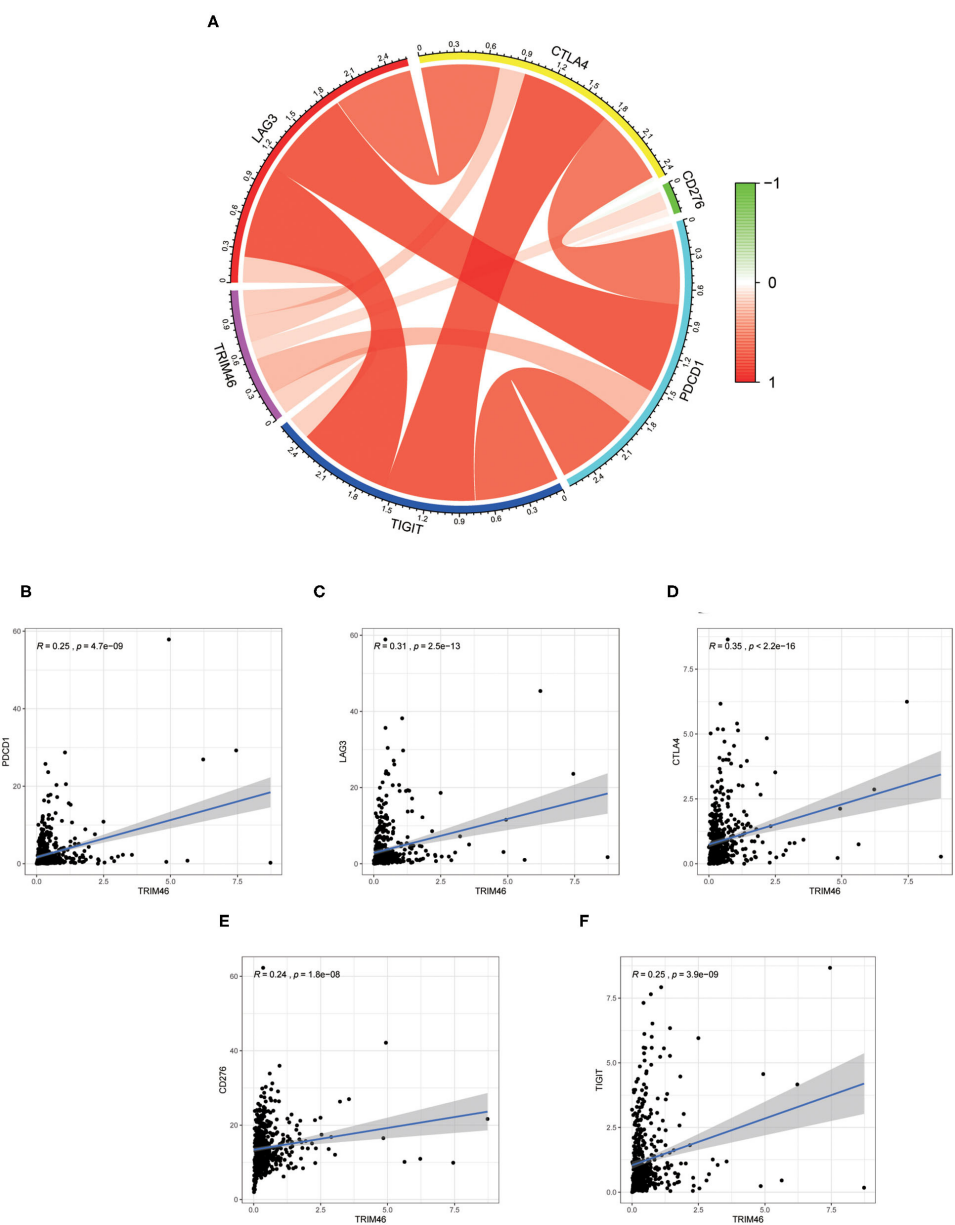
Correlation of the levels of immunosuppressive molecules with TRIM46 expressions. **(A)** Circle diagram of associations between the levels of immunosuppressive molecules and TRIM46 expressions; **(B–F)** TRIM46 was related to TIGIT, CD276, CTLA4, LAG3, and PD-1 according to correlation assays.

Our group observed that TRIM46 was related to the immune levels of CD 4+ T cells (cor = 0.393, *p* < 0.001), macrophages (cor = 0.105, *p* < 0.05), neutrophils (cor = 0.275, *p* < 0.001), and dendritic cells (cor = 0.148, *p* < 0.01) (Figure 9A). Moreover, CIBERSORT analysis indicated that the proportion of activated memory CD4+ T cells (*p* < 0.01) and M0 macrophages (*p* < 0.05) in the TRIM46 high-expression subgroup was significantly higher than that in the low-expression subgroup. Whereas, the proportion of naïve B cell (*p* < 0.05), activated NK cells (*p* < 0.05), eosinophils (*p* < 0.05), and neutrophils (*p* < 0.05) displayed an opposite result (Figure 9B). These altogether indicated that TRIM46 might play an important role in immune cell infiltration in ccRCC progression. Moreover, we evaluate the association between TRIM46 and several immune checkpoints (Figure 10A). The data indicated that TRIM46 was related to DCD1, LAG3, CTLA4, CD276, and TIGIT (Figures 10B–F). Taken together, these results suggested that TRIM46 might cause immunosuppression in ccRCC progression while suggesting that patients with high TRIM46 levels might be more likely to benefit from immune checkpoint inhibitors.

## Discussion

Clear cell renal cell carcinoma is highly heterogeneous in terms of treatment options and clinical outcomes. Metastasis and recurrences are considered to be the main causes for the poor prognosis of patients (14). Moreover, the potential mechanisms involving tumor metastasis remained largely unclear. Frustratingly, unlike most malignancies, ccRCC is insensitive to radiotherapy, and despite significant progress in the study of targeted agents, many patients with the advanced or metastatic disease die due to tolerance to these agents or the drug side effects, resulting in an unsatisfactory overall prognosis (1, 4). Therefore, it is imperative to find novel therapeutic targets for ccRCC and investigate their action mechanisms.

Tripartite motif family proteins are identified as a subfamily of ring E3 ubiquitin ligases, and most of them have E3 ubiquitin ligase activities. Accumulating evidence has demonstrated that TRIM played an important role in various cellular processes, including carcinogenesis, autophagy, innate immunity, protein quality control, apoptosis, developments, and intracellular signaling. Their abnormal expression might result in many diseases such as immunological diseases, developmental disorders, and neoplasms. TRIM46, a member of the TRIM family proteins, was recognized as a crucial regulator of oncogenesis. However, the clinical value and specific effects of TRIM46 in ccRCC were rarely reported.

In the current study, we observed that TRIM46 was significantly elevated in ccRCC specimens. Additionally, we found that TRIM46 overexpression is correlated with poorer clinicopathological features and worse prognosis. Additionally, the elevated expression level of TRIM46 was an independent prognostic factor for poor OS in patients with ccRCC, indicating that TRIM46 may serve as a tumor promotor of ccRCC. Then, we established a nomogram based on the independent prognostic factors in this report, and results showed that this nomogram could efficiently predict the OS of patients with ccRCC.

Next, co-expression analysis identified 10 genes that were strongly associated with TRIM46, including NUMBL, CACNB1, THBS3, ROBO3, MAP3K12, ANKRD13D, PIF1, PRELID3A, ANKRD13B, and PCNX2. Interestingly, these genes were equally overexpressed in ccRCC tissues and their increased levels were associated with advanced clinical stage and worse prognosis. Among them, some are involved in cancer progression. NUMBL was defined as an important regulator in the Notch pathway. Tao et al. (15) found that NUMBL served as a tumor suppressor in glioma and its overexpression inhibited the migration and invasion of tumor cells. It has been pointed out that CACNB1 (also known as CAB1) was overexpressed in gastric and esophageal cancer cells (16). Cristiane et al. (17) reported that high levels of THBS3 were observed in biopsies from patients with metastatic osteosarcoma. In pancreatic cancer, ROBO3 was highly expressed in tumor specimens, and its overexpression promoted cellular growth and invasion *in vivo* and *in vitro* (18). In prostate cancer, MAP3K12 exhibited a positively regulatory effect on the metastasis of tumor cells (19). In cervical cancer, loss of PIF1 suppressed the proliferation, blocked the cell cycle, and promoted apoptosis, and PIF1 deletion promoted the expressions of Caspase-3 and Bax, while suppressing the expressions of Bcl-2 (20). Despite the involvement of some of functional partners of TRIM46 in the cancer process, reports of a link between these genes and ccRCC are lacking.

Next, we found the three most significant biological processes are mitotic cell cycle phase transition, DNA-dependent DNA replication, and cell division, indicating that TRIM46 might be related to cell proliferation, depending on the cell cycle. The three most significant signaling pathways are valine, leucine, and isoleucine degradation; fatty acid degradation; and glycerophospholipid metabolism, suggesting TRIM46 might affect the metabolism of substances in tumor cells and thus be involved in ccRCC progression. Dysregulation of the cell cycle is a significant cause of cancer development and a critical factor in promoting the proliferation of ccRCC cells (21, 22). In ccRCC, changes in metabolic pathways, such as fatty acid metabolism and glycerophospholipid metabolism, modulate tumor energetics and biosynthesis (23, 24). Moreover, GESA results showed the four most significantly enriched signaling pathways enriched in the high TRIM46 expression phenotype are cytokine–cytokine receptor interaction, chondroitin sulfate biosynthesis, taste transduction, and homologous recombination. Cytokine–cytokine receptor interaction is an essential immune signaling pathway as it regulates cytokine interactions and thus the progression of cancer (25). Chondroitin sulfate is a key player in regulating cell development, cell adhesion, proliferation, and differentiation (26). Homologous recombination is a vital biological act in the acquisition of genetic information during cell proliferation (27). Taken together, the above results suggested the important association between TRIM46 and the above pathways in ccRCC progression.

TIMER database results indicated that TRIM46 was related to the immune infiltration levels of CD 4+ T cells, macrophages, neutrophils, and dendritic cells; CIBERSORT analysis indicated that the proportion of activated memory CD4+ T cells, regulatory T cells (Tregs), and M0 macrophages were significantly elevated in the TRIM46 high-expression subgroup, suggesting that TRIM46 might exhibit regulatory effects on immune infiltration. Additionally, we assessed the relationship between TRIM46 and immune checkpoints. Herein, we found that TRIM46 is significantly related to several immune checkpoints, including PDCD1, LAG3, CTLA4, CD276, and TIGIT. PDCD1 (PD-1) was reported to suppress CD8 T cell activation and resulted in depletion (28). TIGIT overexpression was demonstrated to suppress immune cell function at multiple steps (29). Our results, together with previous findings, indicated that TEIM46 promoted ccRCC progression *via* inhibiting the immune system.

Several limitations should be neglected. Firstly, due to the retrospective data and limited clinical information from TCGA, more clinical samples with survival data of long-term survivals were needed to demonstrate our findings. Secondly, the expression of TRIM46 at protein levels in tumor specimens was not reported, which was needed to be further demonstrated. Thirdly, the potential mechanisms involved in the effects of TRIM46 on the clinical outcome of ccRCC patients were not fully explored (concerned with cell proliferation, or metabolic reprogramming, or immune infiltrates, immunosuppression, or both?).

## Conclusion

This study comprehensively explored the clinical value and potential action mechanisms of TRIM46 in ccRCC. Elevation of TRIM46 is considerably associated with ccRCC progression. TRIM46 may be linked to cell proliferation, metabolic reprogramming, immune infiltrates, and immunosuppression of ccRCC. Taken together, TRIM46 can serve as a novel biomarker for diagnosis and prognosis and potential therapeutic target in ccRCC.

## Data Availability Statement

The datasets presented in this study can be found in online repositories. The names of the repository/repositories and accession number(s) can be found in the article/Supplementary Material.

## Author Contributions

All authors made a significant contribution to the work reported, whether that is in the conception, study design, execution, acquisition of data, analysis and interpretation, or in all these areas, took part in drafting, revising, or critically reviewing the article, gave final approval of the version to be published, have agreed on the journal to which the article has been submitted, and agree to be accountable for all aspects of the work.

## Funding

This work was supported by Shandong Medical and Health Science and Technology Development Program (No. 2016WS0446) and Shandong Key Research and Development Plan (No. 2018GSF118189).

## Conflict of Interest

The authors declare that the research was conducted in the absence of any commercial or financial relationships that could be construed as a potential conflict of interest.

## Publisher's Note

All claims expressed in this article are solely those of the authors and do not necessarily represent those of their affiliated organizations, or those of the publisher, the editors and the reviewers. Any product that may be evaluated in this article, or claim that may be made by its manufacturer, is not guaranteed or endorsed by the publisher.
