# Supplementary material for: Identification TRIM46 as a Potential Biomarker and Therapeutic Target for Clear Cell Renal Cell Carcinoma Through Comprehensive Bioinformatics Analyses
Source: Front Med (Lausanne). 2021 Nov 22;8:785331. doi: 10.3389/fmed.2021.785331 (PMC8645697; doi:10.3389/fmed.2021.785331)
Supplement: Supplementary file 1 [file Table_1.DOCX]

**Table S1.** Characteristics of clear cell renal cell carcinoma patients in the TCGA database.

| Clinical parameters | Variables | Total (n=530) | Percentages (%) |
| --- | --- | --- | --- |
| age | ≤60 | 264 | 49.81 |
|  | ＞60 | 266 | 50.19 |
| gender | female | 186 | 35.09 |
|  | male | 344 | 64.91 |
| Histological grade | G1 | 14 | 2.64 |
|  | G2 | 227 | 42.83 |
|  | G3 | 206 | 38.87 |
|  | G4 | 75 | 14.15 |
|  | GX | 5 | 0.94 |
|  | unknow | 3 | 0.57 |
| clinical stage | stage I | 265 | 50 |
|  | stage II | 57 | 10.74 |
|  | stage III | 123 | 23.21 |
|  | stage IV | 82 | 15.48 |
|  | unknow | 3 | 0.57 |
| T classification | T1 | 271 | 51.13 |
|  | T2 | 69 | 13.02 |
|  | T3 | 179 | 33.77 |
|  | T4 | 11 | 2.08 |
| Distant metastasis | M0 | 420 | 79.25 |
|  | M1 | 78 | 14.72 |
|  | MX | 30 | 5.66 |
|  | unknow | 2 | 0.37 |
| Lymph nodes | N0 | 239 | 45.09 |
|  | N1 | 16 | 3.02 |
|  | NX | 275 | 51.89 |

TCGA, The Cancer Genome Atlas.
