# Supplementary material for: Identification TRIM46 as a Potential Biomarker and Therapeutic Target for Clear Cell Renal Cell Carcinoma Through Comprehensive Bioinformatics Analyses
Source: Front Med (Lausanne). 2021 Nov 22;8:785331. doi: 10.3389/fmed.2021.785331 (PMC8645697; doi:10.3389/fmed.2021.785331)
Supplement: Supplementary file 2 [file Table_2.DOCX]

**TableS2**. Co-expressed genes with TRIM46

| gene1 | gene2 | cor | p-value |
| --- | --- | --- | --- |
| TRIM46 | TRIM46 | 1.000 | 0.00E+00 |
| TRIM46 | NUMBL | 0.610 | 2.31E-55 |
| TRIM46 | CACNB1 | 0.597 | 1.52E-52 |
| TRIM46 | THBS3 | 0.577 | 2.31E-48 |
| TRIM46 | ROBO3 | 0.548 | 6.28E-43 |
| TRIM46 | MAP3K12 | 0.543 | 4.80E-42 |
| TRIM46 | ANKRD13D | 0.541 | 1.33E-41 |
| TRIM46 | PIF1 | 0.540 | 2.29E-41 |
| TRIM46 | PRELID3A | 0.528 | 2.41E-39 |
| TRIM46 | ANKRD13B | 0.526 | 5.22E-39 |
| TRIM46 | PCNX2 | 0.524 | 9.75E-39 |
| TRIM46 | KCND1 | 0.521 | 3.35E-38 |
| TRIM46 | RAPGEFL1 | 0.516 | 2.34E-37 |
| TRIM46 | PSRC1 | 0.513 | 7.53E-37 |
| TRIM46 | DCST2 | 0.510 | 2.01E-36 |
| TRIM46 | PPM1N | 0.508 | 4.65E-36 |
| TRIM46 | B3GNTL1 | 0.507 | 5.34E-36 |
| TRIM46 | SLC9A5 | 0.507 | 6.30E-36 |
| TRIM46 | NLGN2 | 0.505 | 1.02E-35 |
| TRIM46 | C21orf58 | 0.502 | 3.34E-35 |
| TRIM46 | GNB3 | 0.502 | 3.20E-35 |
| TRIM46 | CHTF18 | 0.499 | 1.15E-34 |
| TRIM46 | HAUS5 | 0.499 | 1.07E-34 |
| TRIM46 | TROAP | 0.499 | 8.68E-35 |
| TRIM46 | ZNF692 | 0.499 | 1.03E-34 |
| TRIM46 | BRSK1 | 0.498 | 1.49E-34 |
| TRIM46 | CCNF | 0.498 | 1.59E-34 |
| TRIM46 | DGKA | 0.498 | 1.72E-34 |
| TRIM46 | AL157935.2 | 0.496 | 2.60E-34 |
| TRIM46 | ZNF783 | 0.495 | 4.87E-34 |
| TRIM46 | PLCXD1 | 0.491 | 1.62E-33 |
| TRIM46 | PRSS53 | 0.491 | 1.95E-33 |
| TRIM46 | AP4B1 | 0.490 | 2.18E-33 |
| TRIM46 | LPCAT4 | 0.490 | 2.72E-33 |
| TRIM46 | IMPDH1 | 0.489 | 3.56E-33 |
| TRIM46 | RHEBL1 | 0.488 | 4.83E-33 |
| TRIM46 | SLC25A37 | 0.488 | 5.07E-33 |
| TRIM46 | PABPC1L | 0.487 | 6.61E-33 |
| TRIM46 | ARHGAP4 | 0.486 | 7.75E-33 |
| TRIM46 | PARP6 | 0.486 | 7.87E-33 |
| TRIM46 | MICALL2 | 0.485 | 1.32E-32 |
| TRIM46 | REM2 | 0.485 | 1.11E-32 |
| TRIM46 | ARMH1 | 0.484 | 1.89E-32 |
| TRIM46 | LPAR2 | 0.483 | 2.44E-32 |
| TRIM46 | TCIRG1 | 0.482 | 3.96E-32 |
| TRIM46 | C1QTNF6 | 0.478 | 1.50E-31 |
| TRIM46 | GSDMB | 0.478 | 1.31E-31 |
| TRIM46 | STX1A | 0.477 | 2.00E-31 |
| TRIM46 | GPR173 | 0.475 | 3.16E-31 |
| TRIM46 | TONSL | 0.475 | 3.93E-31 |
| TRIM46 | FCHSD1 | 0.474 | 5.49E-31 |
| TRIM46 | EFNA3 | 0.473 | 7.41E-31 |
| TRIM46 | MFSD13A | 0.473 | 6.59E-31 |
| TRIM46 | PBX4 | 0.473 | 6.64E-31 |
| TRIM46 | NPEPL1 | 0.471 | 1.30E-30 |
| TRIM46 | UNC13D | 0.471 | 1.27E-30 |
| TRIM46 | GRAMD1A | 0.470 | 1.77E-30 |
| TRIM46 | TFAP2E | 0.470 | 1.90E-30 |
| TRIM46 | C19orf57 | 0.468 | 2.87E-30 |
| TRIM46 | ARMC12 | 0.467 | 4.42E-30 |
| TRIM46 | JAK3 | 0.467 | 4.45E-30 |
| TRIM46 | KMT5C | 0.467 | 4.60E-30 |
| TRIM46 | TMEM44 | 0.467 | 5.05E-30 |
| TRIM46 | RNF215 | 0.466 | 5.38E-30 |
| TRIM46 | AURKB | 0.465 | 7.40E-30 |
| TRIM46 | IRF3 | 0.465 | 8.13E-30 |
| TRIM46 | KCTD17 | 0.465 | 8.89E-30 |
| TRIM46 | P3H1 | 0.465 | 1.00E-29 |
| TRIM46 | CDCA3 | 0.464 | 1.06E-29 |
| TRIM46 | IFFO1 | 0.464 | 1.13E-29 |
| TRIM46 | PLXNB3 | 0.464 | 1.14E-29 |
| TRIM46 | SH3D21 | 0.464 | 1.29E-29 |
| TRIM46 | TACC3 | 0.464 | 1.18E-29 |
| TRIM46 | OSBPL7 | 0.463 | 1.43E-29 |
| TRIM46 | RIN1 | 0.463 | 1.63E-29 |
| TRIM46 | KIF18B | 0.462 | 2.19E-29 |
| TRIM46 | USF1 | 0.462 | 2.35E-29 |
| TRIM46 | ADAMTS10 | 0.460 | 4.26E-29 |
| TRIM46 | ANKLE1 | 0.460 | 3.55E-29 |
| TRIM46 | FCHO1 | 0.460 | 4.78E-29 |
| TRIM46 | LTB4R | 0.460 | 4.71E-29 |
| TRIM46 | CLK2 | 0.459 | 5.57E-29 |
| TRIM46 | PIDD1 | 0.459 | 4.85E-29 |
| TRIM46 | TICRR | 0.459 | 6.32E-29 |
| TRIM46 | TLE6 | 0.459 | 5.74E-29 |
| TRIM46 | GTPBP3 | 0.457 | 1.14E-28 |
| TRIM46 | RAD9A | 0.457 | 9.36E-29 |
| TRIM46 | RHBDL2 | 0.457 | 1.03E-28 |
| TRIM46 | TNNT1 | 0.457 | 1.11E-28 |
| TRIM46 | CHEK2 | 0.456 | 1.64E-28 |
| TRIM46 | EME1 | 0.456 | 1.45E-28 |
| TRIM46 | DTX2 | 0.455 | 1.72E-28 |
| TRIM46 | MXD3 | 0.455 | 1.89E-28 |
| TRIM46 | ANO8 | 0.454 | 3.02E-28 |
| TRIM46 | CLASRP | 0.454 | 2.34E-28 |
| TRIM46 | ENGASE | 0.454 | 3.02E-28 |
| TRIM46 | FKBP10 | 0.454 | 2.29E-28 |
| TRIM46 | LMBR1L | 0.454 | 2.88E-28 |
| TRIM46 | CFAP45 | 0.453 | 3.97E-28 |
| TRIM46 | SPACA6 | 0.453 | 3.07E-28 |
| TRIM46 | TEPSIN | 0.453 | 3.13E-28 |
| TRIM46 | FBF1 | 0.452 | 5.01E-28 |
| TRIM46 | HJURP | 0.452 | 4.41E-28 |
| TRIM46 | POLD1 | 0.452 | 4.61E-28 |
| TRIM46 | SSPO | 0.452 | 4.91E-28 |
| TRIM46 | STX16 | 0.452 | 4.25E-28 |
| TRIM46 | CENPT | 0.449 | 1.24E-27 |
| TRIM46 | CHFR | 0.449 | 1.22E-27 |
| TRIM46 | MEF2B | 0.449 | 1.23E-27 |
| TRIM46 | MLLT11 | 0.449 | 1.20E-27 |
| TRIM46 | REC8 | 0.449 | 1.18E-27 |
| TRIM46 | STX10 | 0.449 | 1.35E-27 |
| TRIM46 | ADAMTS14 | 0.448 | 1.50E-27 |
| TRIM46 | PHF21A | 0.448 | 1.47E-27 |
| TRIM46 | SPDYA | 0.448 | 1.56E-27 |
| TRIM46 | C1orf35 | 0.447 | 1.98E-27 |
| TRIM46 | DENND4B | 0.447 | 2.07E-27 |
| TRIM46 | HAUS8 | 0.447 | 2.46E-27 |
| TRIM46 | DONSON | 0.446 | 3.07E-27 |
| TRIM46 | KAT2A | 0.446 | 2.59E-27 |
| TRIM46 | WNT10B | 0.446 | 2.76E-27 |
| TRIM46 | FBXO43 | 0.445 | 3.42E-27 |
| TRIM46 | CEP164 | 0.444 | 5.70E-27 |
| TRIM46 | PRKCG | 0.444 | 5.38E-27 |
| TRIM46 | TRMT1 | 0.444 | 4.84E-27 |
| TRIM46 | CPSF1 | 0.443 | 6.70E-27 |
| TRIM46 | SMG9 | 0.443 | 6.79E-27 |
| TRIM46 | NUF2 | 0.442 | 8.75E-27 |
| TRIM46 | NYAP1 | 0.442 | 1.04E-26 |
| TRIM46 | SSBP4 | 0.442 | 1.04E-26 |
| TRIM46 | TMEM79 | 0.442 | 1.05E-26 |
| TRIM46 | APLP1 | 0.441 | 1.16E-26 |
| TRIM46 | BRD9 | 0.441 | 1.13E-26 |
| TRIM46 | C19orf66 | 0.441 | 1.22E-26 |
| TRIM46 | CDK5RAP3 | 0.441 | 1.44E-26 |
| TRIM46 | EFNA4 | 0.441 | 1.13E-26 |
| TRIM46 | IGFLR1 | 0.441 | 1.29E-26 |
| TRIM46 | POLE | 0.441 | 1.43E-26 |
| TRIM46 | VAMP1 | 0.441 | 1.35E-26 |
| TRIM46 | AC010616.1 | 0.440 | 1.76E-26 |
| TRIM46 | CELSR3 | 0.439 | 2.57E-26 |
| TRIM46 | DCAF15 | 0.438 | 3.12E-26 |
| TRIM46 | PDLIM7 | 0.438 | 2.88E-26 |
| TRIM46 | SYNGAP1 | 0.438 | 2.73E-26 |
| TRIM46 | CRYGS | 0.437 | 4.13E-26 |
| TRIM46 | MPP3 | 0.437 | 4.08E-26 |
| TRIM46 | ROM1 | 0.437 | 3.94E-26 |
| TRIM46 | TTC13 | 0.437 | 4.55E-26 |
| TRIM46 | DDX11 | 0.436 | 5.06E-26 |
| TRIM46 | ITGAE | 0.436 | 5.83E-26 |
| TRIM46 | KRI1 | 0.436 | 4.98E-26 |
| TRIM46 | P2RY6 | 0.436 | 5.11E-26 |
| TRIM46 | TBX19 | 0.436 | 5.67E-26 |
| TRIM46 | NUP85 | 0.435 | 6.37E-26 |
| TRIM46 | SPEG | 0.435 | 8.05E-26 |
| TRIM46 | ADM5 | 0.434 | 9.90E-26 |
| TRIM46 | SSC5D | 0.434 | 9.47E-26 |
| TRIM46 | CCDC194 | 0.433 | 1.15E-25 |
| TRIM46 | CFAP73 | 0.433 | 1.31E-25 |
| TRIM46 | KCNN4 | 0.433 | 1.20E-25 |
| TRIM46 | PAFAH1B3 | 0.433 | 1.13E-25 |
| TRIM46 | TTC21A | 0.433 | 1.09E-25 |
| TRIM46 | COLGALT1 | 0.432 | 1.82E-25 |
| TRIM46 | GOLGA6L9 | 0.432 | 1.80E-25 |
| TRIM46 | LOXL3 | 0.432 | 1.79E-25 |
| TRIM46 | PLEKHG4 | 0.432 | 1.70E-25 |
| TRIM46 | TRABD | 0.432 | 1.75E-25 |
| TRIM46 | ZNF26 | 0.432 | 1.64E-25 |
| TRIM46 | ARHGEF1 | 0.431 | 2.47E-25 |
| TRIM46 | CARMIL2 | 0.431 | 2.44E-25 |
| TRIM46 | MSS51 | 0.431 | 2.03E-25 |
| TRIM46 | TMEM145 | 0.431 | 2.15E-25 |
| TRIM46 | FAM193B | 0.430 | 3.34E-25 |
| TRIM46 | KIAA1024 | 0.430 | 2.75E-25 |
| TRIM46 | ADAM11 | 0.429 | 3.63E-25 |
| TRIM46 | B4GALNT4 | 0.429 | 3.63E-25 |
| TRIM46 | P2RX5 | 0.429 | 4.39E-25 |
| TRIM46 | SIRT7 | 0.429 | 3.74E-25 |
| TRIM46 | SYTL1 | 0.429 | 3.61E-25 |
| TRIM46 | UBE2C | 0.429 | 4.13E-25 |
| TRIM46 | KHDC1 | 0.428 | 4.48E-25 |
| TRIM46 | TCF3 | 0.428 | 5.34E-25 |
| TRIM46 | TNK2 | 0.428 | 4.73E-25 |
| TRIM46 | TRAF5 | 0.428 | 4.99E-25 |
| TRIM46 | CENPJ | 0.427 | 6.61E-25 |
| TRIM46 | FLT3LG | 0.427 | 6.00E-25 |
| TRIM46 | HDAC10 | 0.427 | 7.02E-25 |
| TRIM46 | SAMD1 | 0.427 | 6.57E-25 |
| TRIM46 | SLC17A9 | 0.427 | 6.81E-25 |
| TRIM46 | SNRPA | 0.427 | 6.33E-25 |
| TRIM46 | STAT2 | 0.427 | 7.27E-25 |
| TRIM46 | CALML6 | 0.426 | 9.03E-25 |
| TRIM46 | CLEC2D | 0.426 | 8.01E-25 |
| TRIM46 | PCYOX1L | 0.426 | 8.15E-25 |
| TRIM46 | PHF19 | 0.426 | 8.16E-25 |
| TRIM46 | PTPRH | 0.426 | 7.93E-25 |
| TRIM46 | ABCA7 | 0.425 | 1.17E-24 |
| TRIM46 | ARHGEF19 | 0.425 | 1.11E-24 |
| TRIM46 | ATXN7L2 | 0.425 | 1.08E-24 |
| TRIM46 | GABBR1 | 0.425 | 1.23E-24 |
| TRIM46 | MIIP | 0.425 | 1.10E-24 |
| TRIM46 | MOV10 | 0.425 | 1.24E-24 |
| TRIM46 | QPCTL | 0.425 | 1.33E-24 |
| TRIM46 | BICRA | 0.424 | 1.44E-24 |
| TRIM46 | CCDC57 | 0.424 | 1.48E-24 |
| TRIM46 | CCDC88B | 0.424 | 1.60E-24 |
| TRIM46 | COL7A1 | 0.424 | 1.74E-24 |
| TRIM46 | DLG4 | 0.424 | 1.51E-24 |
| TRIM46 | SHC1 | 0.424 | 1.36E-24 |
| TRIM46 | SPRED3 | 0.424 | 1.57E-24 |
| TRIM46 | TTLL3 | 0.424 | 1.70E-24 |
| TRIM46 | ZNF251 | 0.424 | 1.52E-24 |
| TRIM46 | ARHGEF39 | 0.423 | 2.16E-24 |
| TRIM46 | MAST1 | 0.423 | 1.79E-24 |
| TRIM46 | TAZ | 0.423 | 1.92E-24 |
| TRIM46 | ADA | 0.422 | 2.85E-24 |
| TRIM46 | FANCA | 0.422 | 2.82E-24 |
| TRIM46 | NTN5 | 0.422 | 2.98E-24 |
| TRIM46 | ZNF600 | 0.422 | 2.95E-24 |
| TRIM46 | CCDC14 | 0.421 | 3.25E-24 |
| TRIM46 | CPNE7 | 0.421 | 3.38E-24 |
| TRIM46 | INTS6L | 0.421 | 3.82E-24 |
| TRIM46 | IQGAP3 | 0.421 | 3.09E-24 |
| TRIM46 | C7orf61 | 0.420 | 4.27E-24 |
| TRIM46 | KLHL17 | 0.420 | 5.06E-24 |
| TRIM46 | SPC24 | 0.420 | 4.58E-24 |
| TRIM46 | TSPYL2 | 0.420 | 4.66E-24 |
| TRIM46 | ADAM8 | 0.419 | 5.88E-24 |
| TRIM46 | ALKBH6 | 0.419 | 6.90E-24 |
| TRIM46 | FBXL6 | 0.419 | 5.90E-24 |
| TRIM46 | GNB1L | 0.419 | 5.67E-24 |
| TRIM46 | NARF | 0.419 | 6.68E-24 |
| TRIM46 | REEP4 | 0.419 | 5.67E-24 |
| TRIM46 | RELT | 0.419 | 6.66E-24 |
| TRIM46 | RUFY4 | 0.419 | 5.72E-24 |
| TRIM46 | AD000671.2 | 0.418 | 6.91E-24 |
| TRIM46 | AGAP6 | 0.418 | 7.53E-24 |
| TRIM46 | FAM72A | 0.418 | 7.58E-24 |
| TRIM46 | LBHD1 | 0.418 | 7.78E-24 |
| TRIM46 | SFI1 | 0.418 | 8.20E-24 |
| TRIM46 | TNFRSF18 | 0.418 | 7.17E-24 |
| TRIM46 | DDX39A | 0.417 | 9.07E-24 |
| TRIM46 | GRIN2D | 0.417 | 1.12E-23 |
| TRIM46 | MC1R | 0.417 | 1.04E-23 |
| TRIM46 | POFUT2 | 0.417 | 1.08E-23 |
| TRIM46 | PRDM15 | 0.417 | 9.28E-24 |
| TRIM46 | AP1G2 | 0.416 | 1.45E-23 |
| TRIM46 | CCDC130 | 0.416 | 1.20E-23 |
| TRIM46 | DAZAP1 | 0.416 | 1.51E-23 |
| TRIM46 | LAT | 0.416 | 1.49E-23 |
| TRIM46 | LGALS1 | 0.416 | 1.28E-23 |
| TRIM46 | PHKG1 | 0.416 | 1.50E-23 |
| TRIM46 | C1orf131 | 0.415 | 1.77E-23 |
| TRIM46 | FAM189B | 0.415 | 1.59E-23 |
| TRIM46 | ACAP1 | 0.414 | 2.34E-23 |
| TRIM46 | CCNL2 | 0.414 | 2.58E-23 |
| TRIM46 | FNBP4 | 0.414 | 2.18E-23 |
| TRIM46 | WDR62 | 0.414 | 2.13E-23 |
| TRIM46 | ZNF341 | 0.414 | 2.48E-23 |
| TRIM46 | CCDC154 | 0.413 | 2.86E-23 |
| TRIM46 | GIPR | 0.413 | 3.24E-23 |
| TRIM46 | HES7 | 0.413 | 2.86E-23 |
| TRIM46 | IRF9 | 0.413 | 2.77E-23 |
| TRIM46 | KIF2C | 0.413 | 2.67E-23 |
| TRIM46 | TNFAIP2 | 0.413 | 2.91E-23 |
| TRIM46 | TNFRSF25 | 0.413 | 2.80E-23 |
| TRIM46 | U2AF1L4 | 0.413 | 2.92E-23 |
| TRIM46 | UPF3B | 0.413 | 2.78E-23 |
| TRIM46 | AGAP4 | 0.412 | 4.23E-23 |
| TRIM46 | C17orf53 | 0.412 | 3.64E-23 |
| TRIM46 | HSH2D | 0.412 | 3.44E-23 |
| TRIM46 | NFKBIZ | 0.412 | 3.85E-23 |
| TRIM46 | ZBP1 | 0.412 | 3.78E-23 |
| TRIM46 | DOK3 | 0.411 | 5.62E-23 |
| TRIM46 | ERMN | 0.411 | 4.51E-23 |
| TRIM46 | TRAIP | 0.411 | 4.70E-23 |
| TRIM46 | ZNF276 | 0.411 | 4.53E-23 |
| TRIM46 | CDC7 | 0.410 | 6.13E-23 |
| TRIM46 | GOLGA8B | 0.410 | 6.88E-23 |
| TRIM46 | KCNIP2 | 0.410 | 7.13E-23 |
| TRIM46 | LOXL1 | 0.410 | 7.56E-23 |
| TRIM46 | RNF166 | 0.410 | 7.28E-23 |
| TRIM46 | BEST4 | 0.409 | 8.80E-23 |
| TRIM46 | CCDC74A | 0.409 | 9.60E-23 |
| TRIM46 | ENO2 | 0.409 | 9.77E-23 |
| TRIM46 | IL23A | 0.409 | 9.67E-23 |
| TRIM46 | ITGB1BP2 | 0.409 | 9.43E-23 |
| TRIM46 | POLG2 | 0.409 | 8.05E-23 |
| TRIM46 | RFLNA | 0.409 | 9.67E-23 |
| TRIM46 | WFDC3 | 0.409 | 8.41E-23 |
| TRIM46 | ADCK5 | 0.408 | 1.18E-22 |
| TRIM46 | KNTC1 | 0.408 | 1.15E-22 |
| TRIM46 | PKMYT1 | 0.408 | 1.22E-22 |
| TRIM46 | XRCC3 | 0.408 | 1.01E-22 |
| TRIM46 | BASP1 | 0.407 | 1.32E-22 |
| TRIM46 | CEACAM19 | 0.407 | 1.57E-22 |
| TRIM46 | CEP131 | 0.407 | 1.50E-22 |
| TRIM46 | CNIH2 | 0.407 | 1.46E-22 |
| TRIM46 | LRFN1 | 0.407 | 1.37E-22 |
| TRIM46 | PRR19 | 0.407 | 1.30E-22 |
| TRIM46 | PTTG1 | 0.407 | 1.57E-22 |
| TRIM46 | SCNM1 | 0.407 | 1.32E-22 |
| TRIM46 | ZGLP1 | 0.407 | 1.53E-22 |
| TRIM46 | ANKZF1 | 0.406 | 1.81E-22 |
| TRIM46 | ARHGAP33 | 0.406 | 1.99E-22 |
| TRIM46 | KHDC4 | 0.406 | 1.85E-22 |
| TRIM46 | UCN | 0.406 | 1.76E-22 |
| TRIM46 | ARHGAP22 | 0.405 | 2.65E-22 |
| TRIM46 | CCDC163 | 0.405 | 2.58E-22 |
| TRIM46 | MRC2 | 0.405 | 2.46E-22 |
| TRIM46 | NPIPA1 | 0.405 | 2.57E-22 |
| TRIM46 | RFC4 | 0.405 | 2.75E-22 |
| TRIM46 | SNRPA1 | 0.405 | 2.28E-22 |
| TRIM46 | SPATA9 | 0.405 | 2.45E-22 |
| TRIM46 | USP21 | 0.405 | 2.77E-22 |
| TRIM46 | ANKRD23 | 0.404 | 3.09E-22 |
| TRIM46 | CDK3 | 0.404 | 3.19E-22 |
| TRIM46 | DBF4B | 0.404 | 2.97E-22 |
| TRIM46 | DNMT3B | 0.404 | 3.46E-22 |
| TRIM46 | FKBP11 | 0.404 | 3.03E-22 |
| TRIM46 | GCNA | 0.404 | 3.53E-22 |
| TRIM46 | ISG20 | 0.403 | 3.97E-22 |
| TRIM46 | KRT15 | 0.403 | 4.59E-22 |
| TRIM46 | CNTNAP1 | 0.402 | 4.89E-22 |
| TRIM46 | LIG1 | 0.402 | 5.10E-22 |
| TRIM46 | VMP1 | 0.402 | 5.83E-22 |
| TRIM46 | CACNB3 | 0.401 | 6.39E-22 |
| TRIM46 | CPNE1 | 0.401 | 7.29E-22 |
| TRIM46 | PLK1 | 0.401 | 6.60E-22 |
| TRIM46 | SEMA4B | 0.401 | 7.48E-22 |
| TRIM46 | TUBB3 | 0.401 | 7.47E-22 |
| TRIM46 | FBXO8 | -0.401 | 7.30E-22 |
| TRIM46 | PCCA | -0.401 | 6.90E-22 |
| TRIM46 | ATP5PB | -0.402 | 5.34E-22 |
| TRIM46 | TMBIM6 | -0.403 | 3.75E-22 |
| TRIM46 | ACADM | -0.405 | 2.48E-22 |
| TRIM46 | ALDH6A1 | -0.405 | 2.49E-22 |
| TRIM46 | NDUFS1 | -0.405 | 2.16E-22 |
| TRIM46 | ARHGAP24 | -0.406 | 2.11E-22 |
| TRIM46 | ASAH1 | -0.408 | 1.12E-22 |
| TRIM46 | PPARG | -0.408 | 1.21E-22 |
| TRIM46 | ACSL1 | -0.409 | 9.28E-23 |
| TRIM46 | EIF4EBP2 | -0.410 | 6.32E-23 |
| TRIM46 | FDX1 | -0.410 | 6.16E-23 |
| TRIM46 | ACAA2 | -0.411 | 5.53E-23 |
| TRIM46 | MAP7 | -0.411 | 5.49E-23 |
| TRIM46 | C1orf210 | -0.415 | 1.83E-23 |
| TRIM46 | GOT1 | -0.415 | 1.72E-23 |
| TRIM46 | CLCN5 | -0.416 | 1.34E-23 |
| TRIM46 | ITGA6 | -0.416 | 1.32E-23 |
| TRIM46 | PLPP3 | -0.417 | 9.04E-24 |
| TRIM46 | FBXL5 | -0.418 | 8.42E-24 |
| TRIM46 | C11orf54 | -0.420 | 5.04E-24 |
| TRIM46 | IMPA2 | -0.420 | 4.22E-24 |
| TRIM46 | TMEM38B | -0.420 | 4.74E-24 |
| TRIM46 | ADH5 | -0.421 | 3.84E-24 |
| TRIM46 | MYO6 | -0.421 | 3.75E-24 |
| TRIM46 | ABHD6 | -0.422 | 2.64E-24 |
| TRIM46 | CDS1 | -0.422 | 2.94E-24 |
| TRIM46 | SLC25A4 | -0.422 | 2.45E-24 |
| TRIM46 | BTD | -0.423 | 2.11E-24 |
| TRIM46 | DUSP3 | -0.424 | 1.51E-24 |
| TRIM46 | HADHB | -0.424 | 1.64E-24 |
| TRIM46 | EHHADH | -0.426 | 8.89E-25 |
| TRIM46 | ACO2 | -0.428 | 5.64E-25 |
| TRIM46 | TPRG1L | -0.429 | 3.84E-25 |
| TRIM46 | DLD | -0.430 | 2.75E-25 |
| TRIM46 | TSPYL1 | -0.431 | 2.38E-25 |
| TRIM46 | HIBADH | -0.432 | 1.77E-25 |
| TRIM46 | PHYH | -0.434 | 8.59E-26 |
| TRIM46 | PANK1 | -0.436 | 5.26E-26 |
| TRIM46 | RBM47 | -0.437 | 3.85E-26 |
| TRIM46 | ETFDH | -0.438 | 3.17E-26 |
| TRIM46 | CPPED1 | -0.445 | 3.80E-27 |
| TRIM46 | SOWAHB | -0.451 | 5.93E-28 |
| TRIM46 | FAHD1 | -0.455 | 2.13E-28 |
| TRIM46 | MUT | -0.455 | 2.17E-28 |
| TRIM46 | ACAT1 | -0.468 | 3.22E-30 |
| TRIM46 | CAT | -0.468 | 2.86E-30 |
| TRIM46 | CPT2 | -0.474 | 4.76E-31 |
| TRIM46 | TMEM192 | -0.475 | 3.85E-31 |
| TRIM46 | HSDL2 | -0.489 | 3.61E-33 |
| TRIM46 | ECI2 | -0.495 | 4.85E-34 |
